# Association of Senescence Markers with Age and Allograft Rejection in Renal Transplant Recipients

**DOI:** 10.3390/biomedicines12102338

**Published:** 2024-10-14

**Authors:** Peter Vavrinec, Jakub Krivy, Sona Sykorova, Helena Bandzuchova, Zuzana Zilinska, Diana Vavrincova-Yaghi

**Affiliations:** 1 Department of Pharmacology and Toxicology, Faculty of Pharmacy, Comenius University in Bratislava, 832 32 Bratislava, Slovakia; vavrinec@fpharm.uniba.sk (P.V.); jakub.krivy@fpharm.uniba.sk (J.K.); sykorova99@uniba.sk (S.S.); 2 National Transplant Organization, 833 03 Bratislava, Slovakia; helena.bandzuchova@nto.sk; 3 Department of Nephrology and Kidney Transplantations, University Hospital and Comenius University in Bratislava, 833 05 Bratislava, Slovakia; zuzana.zilinska@unb.sk

**Keywords:** kidney transplantation, allograft rejection, age, senescence, p16^INK4a^, p27^kip1^

## Abstract

Background/Objectives: Renal transplantation is the treatment of choice for patients with end-stage renal disease. In the last decade, the number of older renal transplant recipients has significantly increased. However, these patients are at a higher risk of developing post-transplant complications. Therefore, identifying the suitable biomarkers to predict which older patients are at risk of complications is crucial. Cellular senescence could provide insights into the increased vulnerability in this population and guide personalized post-transplant care. Methods: This preliminary study involved biopsies from 25 patients with renal allograft rejection and 18 patients without rejection, further divided into older (50–65 years) and younger (29–40 years) groups. Biopsies were collected at different time points after transplantation, and rejection was classified according to the histological Banff 07 criteria. Additionally, immunohistochemistry for the markers of cellular senescence, p27^kip1^ and p16^INK4a^, was performed. Results: We observed that the number of p27^kip1^-positive glomeruli was higher in the older patients with rejection compared to the younger patients with rejection, and a similar pattern was found in the patients without rejection. However, the number of p27^kip1^-positive tubules was higher in the older patients with rejection compared to the younger patients with rejection, as well as compared to both the older and younger patients without rejection. Tubular p16^INK4a^ expression was not significantly different in the older patients with rejection compared to the younger patients with rejection, and the same pattern was observed in the patients without rejection. However, it was increased in the older patients with rejection in comparison to the older patients without rejection. Conclusions: Our preliminary data suggest the strong potential of both p16^INK4a^ and p27^kip1^ as biomarkers of renal graft rejection, particularly in older renal transplant recipients.

## 1. Introduction

Renal transplantation is the best option for patients with end-stage renal disease (ESRD). Because of the great improvements in medical care quality, the number of elderly people has increased, which means the population affected by ESRD has been rapidly growing [1]. Although both allograft and recipient survival have significantly improved over the last ten years, the increased age of recipients remains a risk factor for allograft rejection. Older recipients often suffer from underlying medical conditions, leading to a higher rate of both graft loss and mortality compared to younger transplant recipients [2,3].

To optimize all aspects of the renal transplant procedure, it is necessary to investigate the mechanisms related to the deteriorating survival of older renal transplant recipients. The survival of renal allografts is directly associated with cellular senescence [4,5,6,7], which is also involved in the ageing process [8]. In senescence, irreversible cell cycle arrest occurs due to the accumulation of stress factors, such as ROS or damaged DNA. Senescent cells lose their functionality and produce inflammatory mediators that spread senescence to surrounding cells and the entire organism. Some relevant regulators of senescence are protein p16^INK4a^ or p27^kip1^, which belong to the group of cyclin-dependent kinase inhibitors (CDKs) [9]. In this brief report, we show the expression of the cellular senescence markers p16^INK4a^ and p27^kip1^ in renal allografts of older and younger renal transplant recipients either with or without allograft rejection up to five years after transplantation.

## 2. Materials and Methods

### 2.1. Study Design, Patient Population, Blood, Urine, and Biopsies Sampling

All investigations were carried out under the principles of the Declaration of Helsinki and under approval of the Ethics Committee of the University Hospital Bratislava. All samples were collected following informed consent. Forty-three patients receiving a kidney transplant were included in the study. The immunosuppressive regimen before transplantation and during the follow-up period was previously described [10,11]. The study included 25 patients with allograft rejection and 18 patients without rejection. We further divided our cohort of patients into the following subgroups based on age: younger (29–40 years) patients with (*n* = 14; all male) and without (*n* = 7; 2 female and 5 male) rejection (w/rj and w/o rj, respectively) and older (50–65 years) patients with (*n* = 11; 3 female and 8 male) and without (*n* = 11; 4 female and 7 male) rejection (w/rj and w/o rj, respectively). Blood and urine samples and renal biopsies were collected at the National Transplantation Organization in Bratislava, Slovakia, at different time points after transplantation (from 7 days to 5 years). Rejection episodes were classified according to the histological Banff 07 criteria at the time of biopsy sampling. All patients were tested for cytomegalovirus (CMV) and for p53 (instead of simian vacuolating virus 40-SW40). In our cohort, all the patients were CMV negative; however, one patient was focally positive for p53, as well as another one, although without distinct intranuclear viral inclusion (both from older patients w/rj group), while one patient from the younger group w/rj revealed minimal p53 positivity.

### 2.2. Immunohistochemistry, Fluorescence Multiplex Immunohistochemistry

Immunohistochemistry for p27^kip1^ and p16^INK4a^ was performed on allografts as follows: The biopsies were cut into 3 μm paraffin sections, dewaxed, and subjected to antigen retrieval by a 15 min incubation in 10 mM citrate buffer, pH 6.0 at 80 °C, for both anti-p27^kip1^ and anti-p16^INK4^ antibodies (Abcam, Cambridge, MA, USA). For p16^INK4a^, a 3-step immunoperoxidase technique was used according to standard techniques. Peroxidase activity was developed using diaminobenzidine and H_2_O_2_ [12]. For p27^kip1^, fluorescent multiplex immunohistochemistry with tyramide signal amplification was performed. The number of positive nuclei for tubules or glomeruli per area was counted for p27^kip1^. For p16^INK4a^, the total staining was expressed as a percentage of positive tubules.

### 2.3. Statistical Analysis

Significance was tested by one-way analysis of variance (ANOVA), followed by Tukey’s post hoc test in the case of normal distribution and by the Kruskal–Wallis test followed by Dunn’s post hoc test in the case of non-normal distribution (SPSS Statistics 25.0 software). The potential relationships between parameters were analysed using Pearson’s parametric correlation test or Spearman’s nonparametric test. A *p* value of <0.05 was considered significant, and all declared changes are significant unless stated otherwise (in case of trends, *p* values are stated). Data are presented as the mean ± standard deviation.

## 3. Results

**Figure 1:**
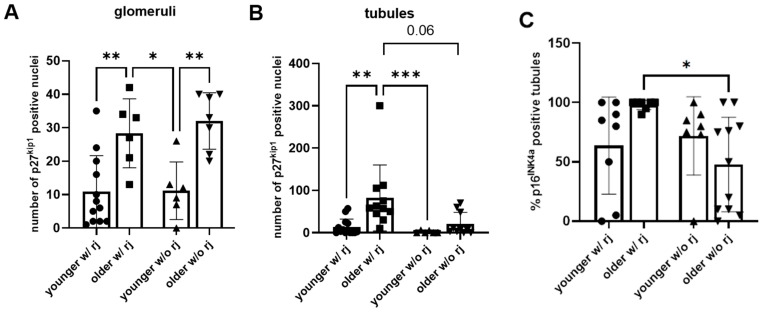

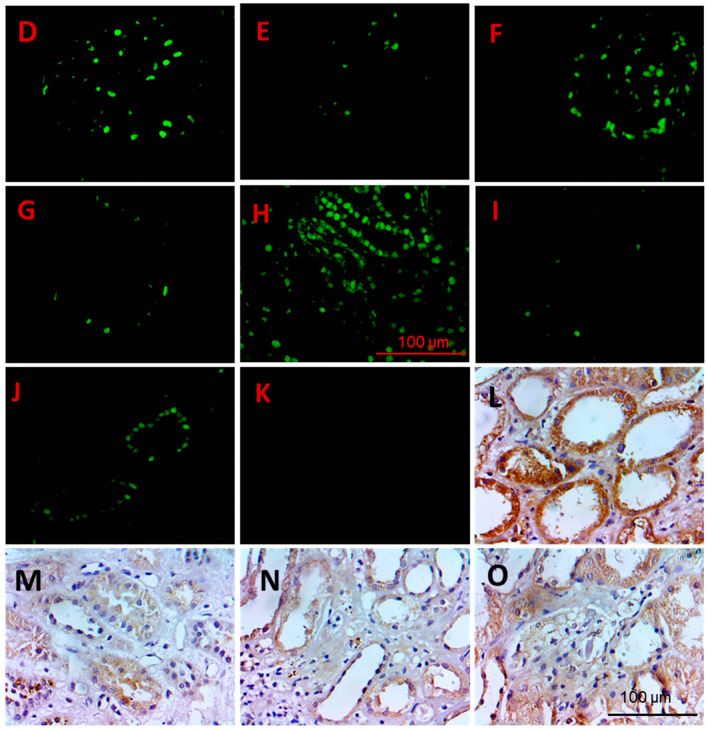
Expression of p27^kip1^ (glomeruli and tubules) and p16^INK4a^ (tubules) in older and younger patients with allograft rejection after kidney transplantation. Localization of p27^kip1^ and p16^INK4a^ proteins in the graft biopsies. (**A**) Glomerular p27^kip1^ expression was increased in older patients compared to younger patients in the group w/rj, as well as in older patients compared to younger patients in the w/o rj group; (**B**) Tubular p27^kip1^ expression was increased in older patients w/rj compared to younger patients w/rj, as well as to both older (*p* = 0.06) and younger patients w/o rj; (**C**) Tubular p16^INKa^ expression was increased in older patients w/rj compared to older patients w/o rj; (**D**) p27^kip1^ expression in the glomeruli of older patients and (**E**) younger patients w/rj and (**F**) older patients and (**G**) younger patients w/o rj. (**H**) Tubular p27^kip1^ expression in older patients and (**I**) younger patients w/rj and (**J**) older patients and (**K**) younger patients w/o rj. (**L**) Tubular p16^INK4a^ expression in older patients and (**M**) younger patients w/rj and (**N**) older patients and (**O**) younger patients w/o rj. Magnification (400×). * *p* < 0.05; ** *p* < 0.01; *** *p* < 0.001; w/rj, with rejection; w/o rj, without rj. Data are presented as mean ± SD.

The number of p27^kip1^-positive glomeruli was higher in the older patients w/rj compared to the younger patients w/rj (Figure 1A). The same pattern was observed in the patients w/o rj (Figure 1A). The number of p27^kip1^-positive tubules was higher in the older patients w/rj compared to the younger patients w/rj (Figure 1B) and compared to both the older and younger patients w/o rj; however, in the case of the older patients w/o rj, the result was on the border of statistical significance (*p* = 0.06, Figure 1B), while no difference was found between the older and younger patients in the group w/o rj (Figure 1B).

Tubular p16^INK4a^ expression was not changed in the older patients w/rj compared to the younger patients w/rj (Figure 1C), and the same pattern was observed in the patients w/o rj (Figure 1C). However, p16^INK4a^ expression was increased in the older patients w/rj in comparison with the older patients w/o rj (Figure 1C). Figure 1D–O depict the representative staining.

The number of p27^kip1^-positive glomeruli showed a trend towards positive correlation with age in the younger patients w/rj (*p* = 0.09; Table 1). A negative correlation with proteinuria was found in the older patients w/o rj (Table 1), while a positive correlation with serum creatinine was found in the younger patients w/o rj (Table 1). The number of p27^kip1^-positive tubules positively correlated with age, cold ischemia, and glomerular filtration rate (GFR) and negatively with serum creatinine in the older patients w/rj (Table 1). In the older patients w/o rj, p27^kip1^-positive tubules correlated positively with age on the border of statistical significance (*p* = 0.06) and negatively with GFR (Table 1). No such correlation was found in the younger patients w/or w/o rj. Tubular p16^INK4a^ expression correlated positively with proteinuria in the older patients w/rj and negatively with age and positively with proteinuria in the younger patients w/rj (Table 1). No such correlation was found in the patients w/o rj.

## 4. Discussion

We observed increased p27^kip1^ staining in the glomeruli of the older patients, regardless of rejection, when compared to the younger ones, also irrespective of rejection. This suggests that glomerular p27^kip1^ expression is connected to age rather than to rejection. In the older group w/o rj, glomerular p27^kip1^ correlated negatively with proteinuria. This is in line with Oka et al., as they found that patients with increased proteinuria showed fewer p27^kip1^-positive podocytes in transplanted kidney and vice versa [13]. However, they found decreased p27^kip1^-positive podocytes and elevated creatinine in two patients with graft rejection [13], which is contrary to our results, as we found increased glomerular p27^kip1^ in both the older w/o and w/rj groups.

p16^INK4a^ is a well-recognized marker of increased creatinine post-transplantation [14] and a key age-related component of senescence in human renal allograft [15], as its expression greatly increases in a transplanted kidney with IF/TA [15]. We found an increase in p16^INK4a^ only in older patients w/rj. In both the older and younger groups, w/rj p16^INK4a^ was associated with proteinuria and, interestingly, in the younger w/rj group, increased p16^INK4a^ was associated with younger age. Further, we observed a substantial increase in p27^kip1^-positive tubules in the older patients w/rj, which reflects both impaired kidney function due to rejection and age. Moreover, only in this group did the tubular p27^kip1^ correlate positively with age, cold ischemia, and GFR and negatively with serum creatinine. Studies exploring p27^kip1^ in renal transplant rejection are scarce. Chkhotua et al. found out that both p27^kip1^ and p16^INK4a^ are age dependent in normal human kidneys, and that they are abundantly increased in chronic allograft nephropathy (CAN), albeit with no correlation with age [16]. In our study, tubular p27^kip1^ correlated with age in the older patients both w/and w/o rj, and glomerular p27^kip1^ as well as p16^INK4a^ correlated with age in the younger group w/rj, but the correlation in this case was negative. The above-mentioned study was, however, conducted in patients of different ages, in the range of 21 to 80 years [16].

It is necessary to consider potentially confounding factors related to our results. Changes in both p27^kip1^ and p16^INK4a^ were reported in many pathological conditions and/or diseases. For instance, p16^INK4a^ is associated with oropharyngeal squamous cell carcinomas [17], human papillomavirus-driven head and neck cancers [18], and lung cancer [19]. Moreover, it was shown that both markers are influenced by COVID-19 infection [20]. All the patients in our cohort were CMV negative and were tested for p53, instead of simian vacuolating virus 40-SW40 which can cause urinary tract and allograft diseases such as hemorrhagic cystitis and polyomavirus-associated nephropathy and is associated with urologic carcinomas in kidney transplant recipients [21]. Only three patients were minimal p53 positive (two from the older patients w/rj group and one from the younger patients w/rj group). Moreover, according to guidelines of the Slovak Republic’s standard procedure of examination of the patient before admission on the waiting list for kidney transplant, it is recommended to exclude candidates with active malignancy from the waiting list. Additionally, patients should undergo testing for human immunodeficiency virus (HIV), hepatitis type B and C, Epstein–Barr virus (EBV), herpesvirus (HSV), varicella zoster virus (VZV), human T-lymphotropic virus type 1 (HTLV), and other infectious diseases such as measles, mumps, and rubella. There were thus no positive cases of such diseases in our cohort of patients. It is important to mention that both transplantations and sampling took place before the COVID-19 pandemic. Based on this information, we can assume that possible bias or artifact results have been eliminated from study. Another cofounding factor is possible gender-related bias. However, although most of our cohort (43 patients) consisted of males, with only nine females, they were more or less equally distributed among the groups.

To the best of our knowledge, this is the first study comparing p27^kip1^ and p16^INK4a^ in older and younger patients w/ and w/o graft rejection. However, further research with a larger number of patients is needed to explore the association of these markers with age and rejection, as well as its diagnostic potential. Moreover, a larger cohort of patients would be important to explore the relationships between the type of rejection (i.e., T-cell mediated vs. antibody mediated, acute rejection vs. CAN) and the above-mentioned parameters. In addition, standardized sampling is also necessary and, most importantly, the analyses of the predictive potential of our markers.

## Figures and Tables

**Table 1:** Correlation analyses of various parameters dependency on glomerular and tubular p27^kip1^ ang glomerular p16^INK4a^ expression.

Protein/Patient Group	Parameter	Correlation Coefficient	*p*-Value
tubular p27^kip1^			
older w/rj	age	0.570	<0.05
	cold ischemia	0.686	<0.05
	serum creat	−0.602	<0.05
	GFR	0.769	<0.05
younger w/rj	no		
older w/o rj	age	0.606	0.06
	GFR	−0.733	<0.05
younger w/o rj	no		
glomerular p27^kip1^			
older w/ rj	no		
younger w/ rj	age	0.507	0.09
older w/o rj	proteinuria	−0.872	<0.05
younger w/o rj	serum creat	0.794	<0.05
glomerular p16^INK4a^			
older w/ rj	proteinuria	0.757	<0.05
younger w/ rj	age	−0.749	<0.05
	proteinuria	0.689	<0.05
older w/o rj	no		
younger w/o rj	no		

Younger patients, 29–40 years; older patients, 50–65 years; w/rj, with rejection; w/o rj, without rejection; creat, creatinine; GFR, glomerular filtration rate; no, no correlation found.

## Data Availability

The data that support the findings of this study are available on request from the corresponding author. The data are not publicly available due to privacy or ethical restrictions.
